# Barriers to Sexual Health Services for Young People in Nepal

**DOI:** 10.3329/jhpn.v28i6.6611

**Published:** 2010-12

**Authors:** Pramod R. Regmi, Edwin van Teijlingen, Padam Simkhada, Dev Raj Acharya

**Affiliations:** ^1^ Section of Population Health, University of Aberdeen, UK; ^2^ Health and Social Care, Bournemouth University, UK; ^3^ Manmohan Memorial Institute of Health Sciences, Purbanchal University, Nepal; ^4^ School of Health and Related Research, University of Sheffield, UK; ^5^ School of Education and Lifelong Learning, Aberystwyth University, UK

**Keywords:** Barriers, Condom, Health education, Health services, Qualitative studies, Sex behaviour, Sexual health, Sexual health services, Nepal

## Abstract

Although sexual and reproductive health education and services are provided to young people, current rates of HIV infection and pregnancy are increasing in Nepal, indicating that young people do not always use sexual health services. Health facilities have apparently failed to provide young people with specialized sexual health education and services. This study explored the barriers to using sexual health services, including condom-use among young people in Nepal. Participants from 10 focus groups and 31 in-depth interviews, carried out by a same-sex researcher, reported many socioeconomic, cultural and physical norms that impose barriers to accessing information on sexual health and relevant services. It is concluded that the establishment of youth-friendly service centres in convenient places might help encourage young people to use sexual health services.

## INTRODUCTION

Sexuality is a fundamental dimension of human life (1), and sexual behaviour of young people is becoming one of the important social and major public-health concerns in recent years (2, 3). As a result of the HIV epidemic, research on sexual behaviours of young people has developed rapidly over the past decades (4–7). It is now widely accepted that sexual and reproductive health issues remain the leading cause of ill-health among young people worldwide and are a growing concern in Nepal (8). Nevertheless, research on young people's sexual health is lacking in Nepal (9, 10).

Sexuality-related topics have largely remained as a taboo in many Asian countries (10–12). Nepalese societies have also many strong traditional norms and beliefs relating to sex and sexuality (13, 14), and these issues are rarely discussed within the family environment. Friendships between girls and boys are still unacceptable in Nepal, and many rural parents even discourage their daughters from meeting or talking with boys. Sexual activities outside marriage are not accepted among the majority of Nepalese societies (15, 16). Despite these traditional views, a significant proportion of Nepalese young people are engaged in pre- and extra-marital sex (10, 17–19). Results of a recent study among college students of Kathmandu showed that about 40% of young men had premarital sex (10). There is a general notion that modernization and globalization have resulted in young people waiting longer before getting married (20–22), and this has created more opportunities for them to spend time in intimate (sexual) relationships before marriage (23) as they spend more years in education and marry later.

It is widely accepted that young people have specific sexual health needs, which vary according to their age, sex, marital and socioeconomic status. When young people engage in unprotected sex, it may result in sexually transmitted infections (STIs) and unintended pregnancies (24, 25). Thus, many health workers around the world are trying to prevent, or at least reduce, risk-taking behaviours of young people (13). However, despite the interest of young people in obtaining relevant information and friendly services (26), the provision of sexual and reproductive health services in Nepal is very inadequate (27),

The Nepalese Government is committed to providing a package of sexual and reproductive health service to young people. The Government has developed the National Adolescent Health and Development Strategy and the Young People Development Programme. These policies have envisaged adolescent and young people as a key target group for integrated sexual and reproductive health services, with interventions planned to increase knowledge on sexual and reproductive health issues and availability of services (27, 28).

Nepalese young people gain information and education on sexual and reproductive health mainly through radio and health-education programmes targetted towards the general population (29). Most sexual and reproductive health services in Nepal are provided through private and public-health centres. These include local pharmacists, public-health practitioners, doctors, nurses, and community health workers. Young people obtain sexual health services when they visit health centres, hospitals, or clinics. However, many such programmes are poorly implemented (28). A very few sexual health services, mainly governmental services in rural areas of Nepal, are available. On the contrary, urban areas have more specialized facilities with many sexual health service centres, which young people can access easily.

The role of private and non-governmental organizations is crucial as they engage young people at a grassroots level in sexual health initiatives. These organizations have mainly focused on service and education, particularly on STIs and HIV/AIDS, family planning, and safe motherhood (29). However, sexual and reproductive health programmes are scattered, and there is a lack of a common forum and coordinating mechanism, which could play a significant role in strengthening the programmes with better output (30). Moreover, many young people do not seek information or services because they think that they are at little or no risk of health problems. Results of a recent study with college students showed that, 48% of sexually-active young people had used condoms during their first sexual intercourse (31). Lack of information, social stigma, and logistical and policy barriers have made it difficult for them to use sexual and reproductive health services (32).

Although young people are provided with limited sexual and reproductive health information and services through different media, rates of HIV infection (33) and teenage pregnancy (34) show that many of them do not frequently use sexual and reproductive health services. Health workers and teachers are reluctant to discuss sexuality and reproductive health issues (28, 35). As the teaching of sexual health is often very poor, it is directly associated with teacher's embarrassment, lack of knowledge, and poor teaching techniques. Teachers are confused as the existing courses are insufficient to address the needs of young people (36, 37). There is also a notion that Nepal's political instability and recent political conflict remains a threat to the delivery of healthcare information and service (38). These findings clearly suggest that young people face different forms of barriers to using sexual health services. This qualitative study aims to explore the major barriers faced by young people to using sexual health information and accessing relevant services in Nepal.

## MATERIALS AND METHODS

### Data-collection tools

The topic being sensitive in Nepal, we adopted a qualitative approach. Over the past two decades, there has been a notable increase in the use of qualitative methods to explore sensitive issues, including sexuality (39). Although combining quantitative and qualitative methods is becoming very popular (40, 41), few have explicitly addressed the implications of combining qualitative data-collection methods (42–44). Lambert and Loiselle argued that combining these methods may help generate complementary views (45). Besides, multiple qualitative methods enhance the analysis of a phenomenon and broaden its conceptualization (43).

In 2007, 10 focus groups and 31 in-depth interviews of individuals were carried out among young people in Nepal. In-depth interviews were conducted to get responses from those participants who were either unable or unwilling to attend the focus groups. It was also intended to explore more personal experiences from the participants.

A Nepalese version of the questioning route (46, 47) was designed for the focus groups. It is assumed that the questioning route approach helps provide consistent information, improves the comparability of information among groups, and overcomes the need for a moderator to formulate unprepared questions (46). Similarly, a topic guide was used for conducting semi-structured in-depth interviews. A same-sex researcher conducted focus groups and in-depth interviews as, in less-developed settings, the interviewer's gender does have a significant influence on responses to sensitive questions (48). All focus groups and interviews were conducted in Nepali in a comfortable environment, i.e. a closed room to assure confidentiality. They were tape-recorded with participants' permission and generally lasted for 1–2 hours (49).

### Study areas and participants

Participants for the study were selected purposively (50) from major urban and rural areas of Kathmandu and Chitwan districts. We selected four colleges and one youth club from Kathmandu and three colleges and two youth clubs in Chitwan district. Most focus-group and interview participants were aged 18–22 years. Educated participants were gathered with the help of colleges/universities whereas school/college drop-out participants were found with the help of communities and local youth clubs. Both college-based and school drop-out focus groups were largely drawn from pre-existing groups, i.e. members of the focus groups already know each other. One advantage of using a pre-existing group is that they may feel more at ease taking an active part in the discussion and/or contradicting each other (7, 51). Hennink argues that the composition of pre-existing groups can generally promote debate among group members and enrich the discussion (46). However, there is also a fear that familiarity between participants can also lead to group members being afraid of being challenged by other group members due to the shared knowledge of their experiences. Finally, the research team checked participants for their eligibility. Those who fulfilled the eligibility criteria, such as residence (urban-rural), age (15–24 years), education (school going/school drop-out), and sexual history, were invited to participate in the study.

### Coding and analysis

Transcriptions were made based on the original tape-recordings (52). The completed transcription was compared with hand-written notes to fill in inaudible phrases or gaps in the transcription. Data were organized using the NVivo software (version 8) (QSR International, Southport, UK). One of the main advantages of applying the NVivo software is its ability to ensure the processes of coding; so, the management of data becomes more visual and more flexible (53). A thematic approach was used for analyzing data using categories (or themes or codes) from the dataset (54, 55). Relevant quotes are also provided in the text to illustrate these categories.

### Ethics

Ethical approval for the study was granted by the Nepal Health Research Council, and consent was taken from participants before conducting the study.

## RESULTS

The following themes were identified in the data: (a) embarrassment and poor negotiation skills; (b) poor youth-friendly services; (c) poor knowledge on sexual and reproductive health; and (d) influence of alcohol and the role of peers.

This paper uses the terms ‘young women’ and ‘girls’ and also ‘young men’ and ‘boys’ interchangeably. We are aware that there is a difference between the terms but this is the way the young people in our studies were portrayed.

### Embarrassment and poor negotiation skills

The participants felt embarrassed while talking about sexual and reproductive health matters with friends of the opposite sex, family, and even with their sexual partners. They thought that girls found it difficult to initiate a discussion about it with their sexual partners. Boys argued that it was difficult to keep condoms as they felt embarrassed when caught by family or friends.

We cannot keep condoms in our pocket because, if anyone knew about it [condom], it is not taken positively. When we get the opportunity, we cannot arrange condoms at that time as we do not have time for that. We do [sex] without it [condom] (a rural married male aged 23 years).

Most unmarried boys and girls felt shy and uncomfortable while buying condoms and other contraceptives from local stores, although urban participants claimed that the large number of urban stores provide an opportunity to them to buy condom anonymously. Rural boys argued that the local shopkeepers and health service providers know them and their family; hence, they fear service providers who may share information about them with their friends and family members. This leads to stigmatization, and they may feel too embarrassed to visit health service centres. They also argued that such embarrassment also appeared with doctors or nurses in sexual health clinics.

We know that condoms hould be used but we cannot buy these easily. Even if you go to a shop for this purpose, the shopkeeper looks at you differently (urban females of focus group).

We have a belief that doctors may ask different questions. We always feel fear when answering these questions; so, we rarely go to them [clinics]. We especially feel too shy to share our sexual behaviours with those doctors (rural married females of focus group).

However, some claimed that they would visit the clinic, if there is a service provider of the same gender. They also believed that young people might share their problems with younger service providers.

I am not aware of such services but we may feel more comfortable if there are young service providers. We cannot show parts of our body to male doctors (laughter) (a female school drop-out of focus group).

I do not think that most young people go there for services because there are a very few young service providers. How can we express our feelings to the people who are similar to our parents' age? (an urban unmarried male aged 21 years).

Most clinics were thought to have limited integrated health-related services while other clinics offer sexual health services. In such cases, the respondents were more concerned about being recognized in the clinic by someone whom they knew already.

These services are delivered only in specific places and times. If you appear there at that time, people think that you have some sex-related problems (rural males of focus group,).

Most participants agreed that young people, especially girls, have poor negotiation and decision-making skills which sometimes lead to unsafe sex. Girls further reported that it is not easy to talk about sexual matters with their boyfriends. They concluded that there is less chance of refusing sex if boys ask explicitly.

We cannot wait long for that [sex]. We always rush for fun. When we get the opportunity, we cannot control ourselves. When he sees me, he always expects that [sex] (laughter). I cannot decide what is right and what is wrong (an urban unmarried female aged 22 years).

### Poor youth-friendly services

The urban participants reported that there are many sexual health clinics in town while the rural participants mentioned that they are aware of availability of few health services in the rural areas.

I really do not like to go there [health post] because I know that I cannot get all services there. If we have a serious problem, we go to the hospitals in the town because we know that health post staff eventually transfer us there (a rural married male aged 23 years).

They reported that young people have to rely on these health posts. Most rural participants also highlighted that rural health posts do not provide youth-friendly services. The participants argued that rural young people rarely visit health posts to seek sexual health services. However, they reported that they go there to seek other health-related services. Some participants also reported that, if the sexual health problems become serious, only then they visit health posts. Most participants believed that service providers at health posts do not keep information confidential and do not behave nicely if sexual health problems are shared with them.

Once, I had a pain in my *younanga* [penis]. My friend advised me to visit the health post. When I dared to share my problem, the health service provider shouted at me. Later, I went to another hospital for check-up (a rural unmarried male aged 18 years).

Sexual health services were perceived to be neither sufficient nor youth-friendly. The participants stressed the need for youth-friendly services in all the areas. The rural participants often reported that condoms were not easily available in the rural areas, and they were concerned about other people watching them while buying condoms. The rural participants argued that only a few shops provide condoms, and most rural people know them and their family, which hinders them from buying it.

We have one health post there, and it is very far. The doctors in this health post know almost everybody. We know that we can get condoms from there but we do not visit because we feel too embarrassed to get condoms. Some people even try to get condoms from the hospital but the stocks are not maintained regularly (rural males of focus group).

On the contrary, urban young men shared that they can easily buy condoms. However, the fear of being recognized is still a concern for them. Financial constraints have emerged as a barrier to user-friendly sexual health services for both rural and urban participants. They argued that fees in the private clinics are very high and, as such, young people do not always find it reasonable to access services.

We are satisfied with services provided by the centres but the fee was very high …. We had to pay NRs 3,000 for that [abortion] (an urban unmarried female aged 22 years).

### Poor sexual and reproductive health knowledge

Most boys and girls reported that rural youths have poor sexual health knowledge, which in turn leads to poor sexual health-service utilization. Most boys reported that many young people perform sex; however, there is a notion among the participants that a very few engage in safe sexual practices. Poor knowledge of young people about sexual and reproductive health and poor accessibility of sexual health services forced them to engage in unsafe sexual practices.

I think, in most cases, boys initiate for that [sex], and most of us have poor knowledge about it. Some girls start sex at the age of 15/16 years …. I do not even remember when I started …. 15/16 years? (a female in the school drop-out focus group).

I cannot remember when I did it first time (laughter). When I shared it with my friends, they told me that I might have AIDS. I had heard of some advertisements; so, I was not serious about it, although I was very scared at that time (a rural unmarried male school drop-out aged 18 years).

Friends and the media, such as newspapers, radio, and television, were the main reported sources of information about sexual matters. The participants recognized that their sources of information sometimes might be wrong, and this could lead to confusion and increased vulnerability.

They also stated that some rural parents prefer child marriage, which is part of their culture. These cultures also involve boys and girls engaging in unsafe sex since they are not mature enough in their decision-making.

Some rural parents marry off their children at early ages like 14–15 years. We can easily imagine what they do at that age? This is the time for education but they are deprived of it and are busy with other household chores. They become physically poor and even give immature birth. This all happens because of unsafe sex (a rural unmarried male aged 18 years).

### Influence of alcohol and peers

Most participants stated that alcohol affects sexual behaviours of young people and might lead to poor decision-making.

We did not use a condom. We could not even remember it. I do not know what had happened (a rural married female aged 23 years).

Most of the time, the environment creates the favourable situation for sex. If you are drunk, you feel brave enough to propose sex. You may even force your sex partner because you do not know what you are doing (an urban married male aged 23 years).

Most young men agreed that proposing sex when you are drunk is very easy, and there is less chance of being denied if women are also drunk. Interestingly, urban women also agreed that drinking alcohol at parties is not uncommon and believed that it is also important for socialization. Young men stated that these habits make them feel more confident but their decision towards safer sex could be affected. Young women also shared that alcohol could influence their decision-making.

Many people may get involved in unsafe sex …. I know about safe sex but I could not even remember since we were drunk (an unmarried rural female aged 17 years).

We also found the role of peers to be an important factor in making decisions on alcohol, drugs, sex, and romantic relationship. Most young people in this study who were found to have smoking and drinking habits were influenced by their peers. The participants further explained that young people seek sexual health advice from their friends first. Sometimes, they receive incorrect information from their peers.

## DISCUSSION

Most young people felt embarrassed talking about sexual health (services) with parents, relatives, and senior community members; such embarrassment is, of course, not unique to Nepal (7, 56). Rural young people are more likely to be embarrassed accessing such services than are urban young people since there is a fear of stigmatization from local people in the rural areas. Such embarrassment can also be observed among Nepalese sex workers accessing sexual health services since they are worried about being identified as sex workers by clinic staff (57). Our findings indicate that the young people of Nepal fear sexual health service providers to be judgemental and lack confidentiality. Several studies show that young people choose not to access sexual health services because they perceive clinic staff to be judgemental and lacking confidentiality (58, 59). Evidence also showed the effectiveness of respecting young people's confidentiality in preventing teenage pregnancy (60).

Our findings suggest that negotiation and decision-making skills are necessary for these young people to abstain from unprotected sexual practices (61). However, many Nepalese young people lack such skills. Jha and colleagues found that many young people in Nepal lack the power and skills to use sexual and reproductive health services (8). Hence, programme designers should also focus on developing negotiation skills in young people.

Youth-friendly services for young people have a significant role in disseminating sexual health information and services (37, 62). In the contemporary Nepal, many rural young people rely on government health services, which open at the same time as schools and colleges. In such situations, young people need to be absent from school/college if they require some sort of sexual health information and services. Such barriers to using sexual health services have also been reported from many developed countries. Opening hours for many clinics were identified as a barrier for young people in the UK (59) and in Africa (32, 58). This suggests the opening of the sexual health clinics during weekends and holidays for school- or college-going adolescents and young people. Establishment of such service centres in convenient places and time would encourage young people to visit the service centres. Similarly, providing essential materials, such as condoms, educational leaflets, booklets, and pamphlets through the youth-friendly service centre would encourage young people to use these services frequently.

Nepal is one of the poorest countries in the world, and the economic constraints of the young people affect their ability to buy contraceptives or seek sexual and reproductive health services (22, 24). For most young couples, economic issues play a central role in sexual healthcare decision-making. High costs associated with pregnancy-related healthcare services result in lower participation by women in decision-making about antenatal services in Nepal (63). This suggests that clinics for sexual health service should be free or at a discounted charge as many young people do not have enough money to spend on their healthcare. A study in Africa reported that poverty was a powerful agent in preventing young people from purchasing condoms (64).

Studies in Nepal have documented that knowledge about sexual and reproductive health, particularly about STIs and condoms, is inadequate and that knowledge among male and urban young people was generally better than females and rural young people (8, 9, 65). Poor knowledge about many aspects of sexual health is unlikely to encourage the use of sexual and reproductive health services. Interestingly, awareness of HIV/AIDS is higher than that of other STIs (65, 66). Perhaps this must be considered a success as dissemination of information on HIV/AIDS is a relatively new phenomenon in Nepal, advocated widely only in the previous decade. This suggests that mass media would be a possible means of disseminating information on sexual health to urban youths. Sexual health education through trained peer educators could be an effective method of improving the knowledge of young people on the issues of sexual and reproductive health (67).

The use of drugs and alcohol creates a barrier to accessing sexual health services among young people while it encourages involvement in unsafe sex. Previous studies with Nepalese young people and Nepalese trekking guides also documented similar findings (14, 31, 68, 69). We suggest that young people and the community as a whole need to be better informed of the serious negative sexual health consequences of smoking and drinking.

### Limitations

This study had several limitations. The study was conducted in two districts, namely Chitwan and Kathmandu; hence, it is difficult to generalize our findings across other areas of the country. Being a multi-cultural and multi-ethnic society, different cultures and ethnic groups of Nepal have their own norms and values around sexuality. However, we could not analyze our data based on ethnicity and religion. Finally, although it is widely accepted that sex and sex-related issues are not openly discussed in Nepal, most young people in this study actively discussed sex-related issues. It can be argued that many sexual and reproductive health programmes in the media encouraged them to participate in our study. Having researchers of the same sex as the participants for the focus-group discussions and interviews and assuring confidentiality may also have contributed to the good response from young people.

### Conclusions

The key barriers to the use of sexual health services in Nepal do not vary greatly from both developed and developing countries as revealed in similar studies. What seems to be needed is a sexual health service which maintains confidentiality, treats young people with respect, and ensures that their voices are heard. They should be provided with all the necessary negotiation skills to avoid the future risks of HIV/STIs and unwanted pregnancy. Perhaps, free and discounted sexual health services from governmental, non-governmental and community-based organizations would motivate young people to use these services. Similarly, incorporating peer-education programmes on sexual health through educational and community-based organizations and the establishment of youth-friendly service centres in convenient places and time would help encourage young people, especially those living in rural areas, to use sexual health services more frequently.

## ACKNOWLEDGEMENTS

This study was supported by a grant to the first author from the University of Aberdeen and the Carnegie Trust for Universities of Scotland.

The authors thank all participants in Nepal and the interviewers and focus-group facilitators. They are thankful for the useful comments made by the reviewers on their initial submission.
